# *In vivo* efficacy of the AKT inhibitor ARQ 092 in Noonan Syndrome with multiple lentigines-associated hypertrophic cardiomyopathy

**DOI:** 10.1371/journal.pone.0178905

**Published:** 2017-06-05

**Authors:** Jianxun Wang, Vasanth Chandrasekhar, Giovanni Abbadessa, Yi Yu, Brian Schwartz, Maria I. Kontaridis

**Affiliations:** 1 Department of Medicine, Division of Cardiology, Beth Israel Deaconess Medical Center, Boston, Massachusetts, United States of America; 2 ArQule, Inc., Burlington, Massachusetts, United States of America; 3 Department of Biological Chemistry and Molecular Pharmacology, Harvard Medical School, Boston, Massachusetts, United States of America; Albert Einstein College of Medicine, UNITED STATES

## Abstract

Noonan Syndrome with Multiple Lentigines (NSML, formerly LEOPARD syndrome) is an autosomal dominant "RASopathy" disorder manifesting in congenital heart disease. Most cases of NSML are caused by catalytically inactivating mutations in the protein tyrosine phosphatase (PTP), non-receptor type 11 (*PTPN11*), encoding the SH2 domain-containing PTP-2 (SHP2) protein. We previously generated knock-in mice harboring the *PTPN11* mutation Y279C, one of the most common NSML alleles; these now-termed SHP2^Y279C/+^ mice recapitulate the human disorder and develop hypertrophic cardiomyopathy (HCM) by 12 weeks of age. Functionally, heart and/or cardiomyocyte lysates from SHP2^Y279C/+^ mice exhibit increased basal and agonist-induced AKT and mTOR activities. Here, we sought to determine whether we could reverse the hypertrophy in SHP2^Y279C/+^ mice using ARQ 092, an oral and selective allosteric AKT inhibitor currently in clinical trials for patients with PI3K/AKT-driven tumors or Proteus syndrome. We obtained echocardiographs of SHP2^Y279C/+^ and wildtype (SHP2^+/+^) littermates, either in the presence or absence of ARQ 092 at 12, 14, and 16 weeks of age. While SHP2^Y279C/+^ mice developed significant left ventricular hypertrophy by 12 weeks, as indicated by decreased chamber dimension and increased posterior wall thickness, treatment of SHP2^Y279C/+^ mice with ARQ 092 normalized the hypertrophy in as early as 2 weeks following treatment, with hearts comparable in size to those in wildtype (SHP2^+/+^) mice. In addition, we observed an increase in fractional shortening (FS%) in SHP2^Y279C/+^ mice, an effect of increased compensatory hypertrophy, which was not apparent in SHP2^Y279C/+^ mice treated with ARQ 092, suggesting functional improvement of HCM upon treatment with the AKT inhibitor. Finally, we found that ARQ 092 specifically inhibited AKT activity, as well as its downstream effectors, PRAS and S6RP in NSML mice. Taken together, these data suggest ARQ 092 may be a promising novel therapy for treatment of hypertrophy in NSML patients.

## Introduction

Noonan Syndrome with Multiple Lentigines (NSML) (MIM151100), formerly known as LEOPARD syndrome, belongs to a family of autosomal dominant “RASopathy” disorders that are caused by mutations in components of the RAS/MAPK pathway [1–3]. NSML typically presents with multiple phenotypic abnormalities, including multiple lentigines on the skin, electrocardiographic conduction abnormalities, ocular hypertelorism, pulmonic stenosis, abnormal genitalia, retardation of growth, and sensorineural deafness [4]. In addition, and the most common and deleterious hallmark of this disease, is the presence of congenital heart defects (CHDs), and in particular, hypertrophic cardiomyopathy (HCM), an abnormal thickening of the heart that eventually leads to cardiac functional abnormalities and heart failure [5–9]. NSML is almost exclusively caused by catalytically inactivating mutations in the protein tyrosine phosphatase (PTP) non-receptor type 11 (*PTPN11*) gene that encodes the SH2 domain-containing PTP-2 (SHP2) protein [3,10]. We previously generated knock-in mice harboring the *PTPN11* mutation Y279C (hereafter SHP2^Y279C/+^ mice), one of the most common NSML mutant alleles in SHP2 [11]. These SHP2^Y279C/+^ mice recapitulate the human disorder, with short stature, craniofacial dysmorphia, and morphologic, histologic, echocardiographic, and molecular evidence of HCM [11,12]. Heart and/or cardiomyocyte lysates from adult SHP2^Y279C/+^ mice show enhanced binding of SHP2 to IRS1, decreased SHP2 catalytic activity, and abrogated agonist-evoked ERK/MAPK signaling [11]. SHP2^Y279C/+^ mice also exhibit increased basal and agonist-induced AKT and mTOR activity [11,12]. Importantly, the inhibition of mTOR with rapamycin reverses the HCM phenotype in these mice [11]. Here, we sought to determine whether the cardiac defects in SHP2^Y279C/+^ mice could be reversed by treatment with the upstream regulator of mTOR, AKT, by using a novel AKT inhibitor (ARQ 092) currently in clinical trials for patients with PI3K/AKT-driven tumors and Proteus syndrome [13]. Data from our studies would suggest that inhibition of AKT may also be an effective treatment strategy for patients with NSML-associated HCM, and perhaps also in other, more common, CHD-associated HCM diseases.

## Results

### ARQ 092 treatment normalized AKT/mTOR activity in NSML mice

**Fig 1:**
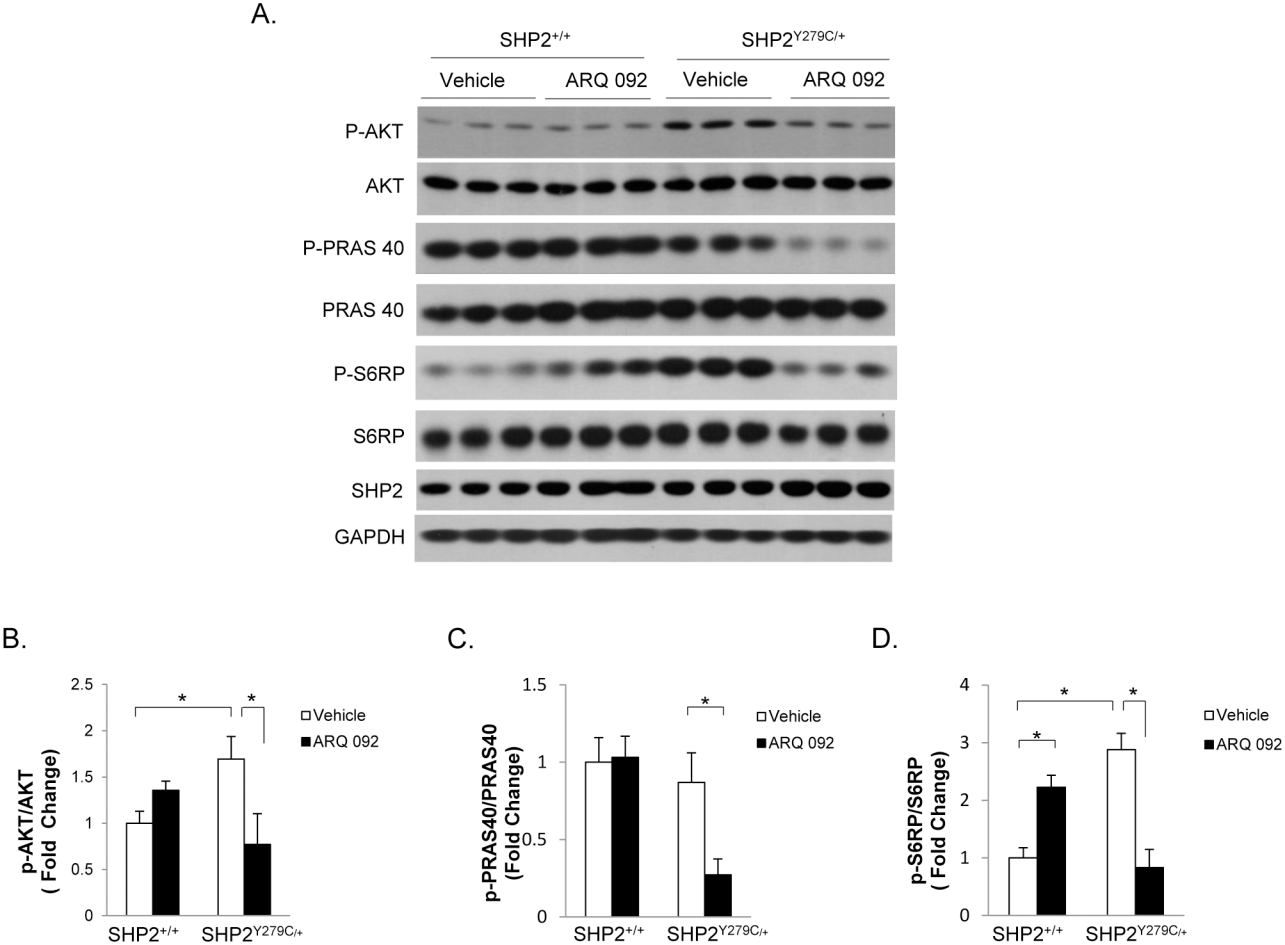
ARQ 092—Treated SHP2^Y279C/+^ hearts have normalized AKT/mTOR signaling. Mouse heart lysates were generated from wild-type (SHP2^+/+^) and NSML (SHP2^Y279C/+^) littermates subjected to either vehicle or ARQ 092 treatment (100mg/kg/day orally) for 4 weeks, starting at 12 weeks of age. The heart lysates were immunoblotted with anti—phospho-AKT, anti—phospho-S6RP and anti-phospho-PRAS40, as indicated, followed by anti-AKT, anti-S6RP, and anti-PRAS40, respectively, to control for loading. Parallel experimental samples were blotted with anti-SHP2 or anti-GAPDH to determine expression levels of these proteins. Quantification of data (n = 3–7 mice/group) is shown to the right of each representative Western blot. Data represent mean ± SEM; *P < 0.05, 2-way ANOVA on ranked data, with Bonferroni post hoc test when ANOVA was significant.

The AKT/mTOR pathway is hyperactivated in SHP2^Y279C/+^ hearts [11,12]. SHP2 also plays a significant, although complex, role in PI3K/AKT pathway regulation [14–16]. To determine the effects of AKT inhibition specifically on SHP2^Y279C/+^ -associated hypertrophy, we isolated whole hearts from 16-week old SHP2^Y279C/+^ and SHP2^+/+^ mice that were treated for 4 weeks (starting at 12 weeks of age) with either vehicle or ARQ 092 (100mg/kg/day), which is in clinical trials for patients with PI3K/AKT-driven tumors or Proteus syndrome [13]. Our experimental dose herein was selected based on maximum tolerable dose (MTD) studies previously conducted at ArQule, Inc., where 100mg/kg/day was found to be both sub-MTD and efficacious in a long-term study [13]. No mortality occurred in our cohort as a consequence of treatment with the AKT inhibitor at this dose. At baseline, pAKT (S473) phosphorylation was significantly increased in vehicle-treated SHP2^Y279C/+^ heart lysates [Fig 1A and 1B and [11,12]]. However, in response to 4 weeks of oral gavage treatment with ARQ 092, we found that pAKT (S473) activity was normalized in SHP2^Y279C/+^ hearts, to levels similar to those of SHP2^+/+^ (Fig 1A and 1B). Interestingly, Proline-rich AKT1 substrate 1 (PRAS), a substrate for AKT that relieves the inhibitory function on mTOR, did not have altered activity in SHP2^Y279C/+^ hearts (Fig 1A and 1C); despite this, PRAS activity was significantly reduced in response to ARQ 092 treatment in NSML hearts (Fig 1C). In contrast, pS6RP, a direct downstream target of mTOR, was hyperactivated in SHP2^Y279C/+^ hearts (Fig 1A and 1D), suggesting that modulation of this pathway is significantly altered in NSML and that treatment with ARQ 092 normalizes AKT/mTOR activity in SHP2^Y279C/+^ mouse hearts.

### Inhibition of AKT signaling normalizes cardiac hypertrophy in NSML mice

**Fig 2:**
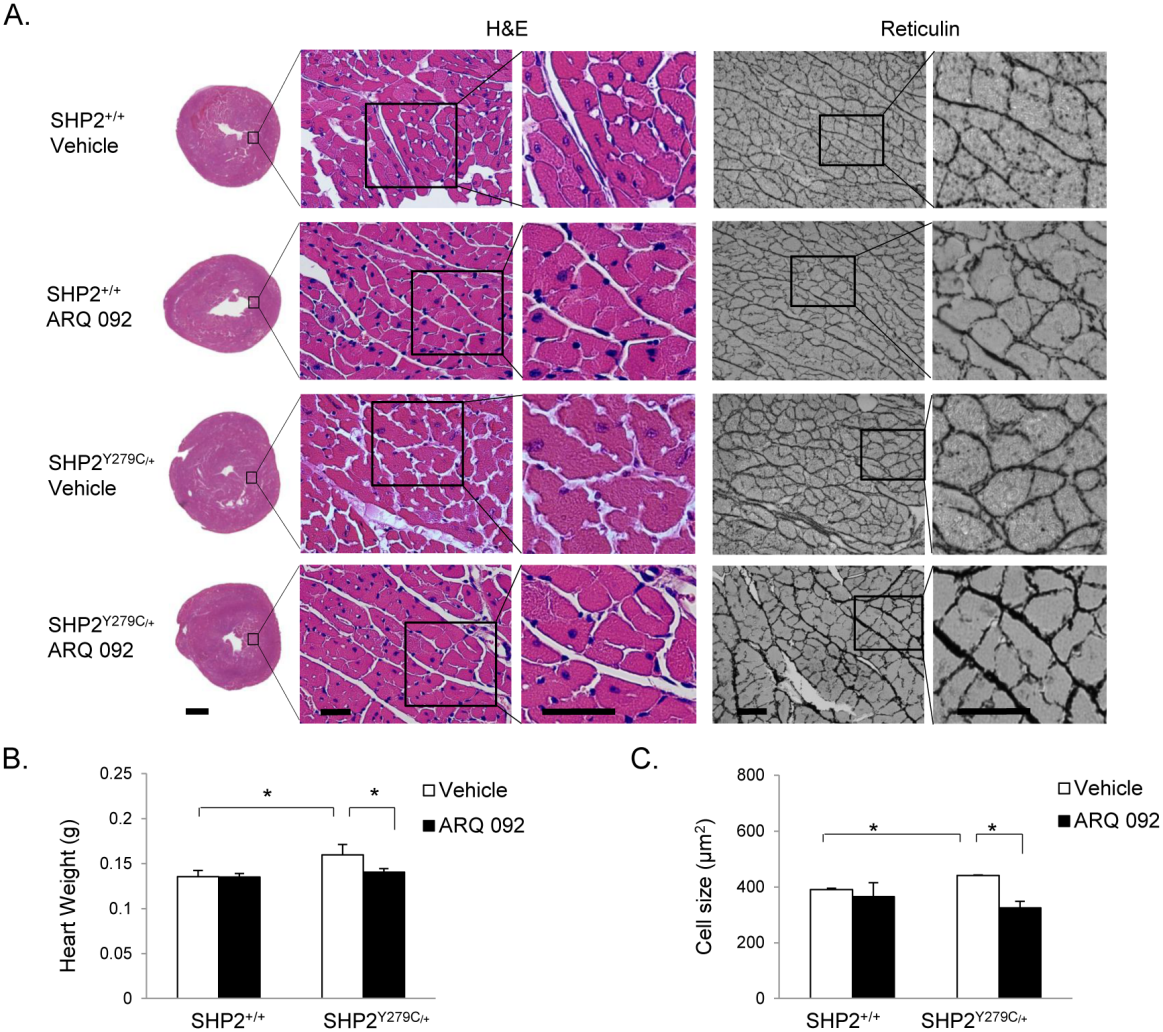
Treatment with ARQ 092 reduces cardiac hypertrophy and normalizes heart size in NSML. 16 week-old SHP2 wild-type (SHP2^+/+^) and NSML (SHP2^Y279C/+^) littermates were treated with either vehicle or ARQ 092 AKT inhibitor (of 100mg/kg/day orally) for 4 weeks, beginning at 12 weeks of age. (A). Representative transverse cross-section of whole hearts (scale bar 1mm) and H&E- and Reticulin-stained sections (scale bar 50 microns); enlarged inset images represent the indicated areas (scale bar 50 microns). Total heart weight (B) and cardiomyocyte cell size (C), as measured from reticulin-stained heart cross-sections. *P < 0.05, where P values were derived from 2-way ANOVA on ranked data, with Bonferroni post hoc test when ANOVA was significant; n = 3–7 mice/group.

To determine the effects of ARQ 092 treatment on cardiac physiology and to assess its effects in cardiac hypertrophy specifically in NSML mice, we conducted a series of histological analyses. As previously described [11,12], H&E-stained transverse sections of 16-week-old SHP2^Y279C/+^ hearts revealed an enlarged and thickening heart, as compared to SHP2^+/+^ littermate controls (Fig 2A). In response to treatment with ARQ 092 for 4 weeks, however, NSML hearts showed a normalized morphology that was similar to that of SHP2^+/+^ hearts. Indeed, overall heart weight in NSML hearts was normalized to weights similar to those in SHP2^+/+^ hearts (Fig 2A and 2B); however, heart weight to body weight and heart weight to tibia length ratios did not reach significance in our experiments, likely due to the dip in body weight in the first 2 weeks in response to the ARQ 092 treatment and the disproportionate effects of bone structure to body length ratios in NSML mice, respectively, that might confound those ratio measurements [S1 Fig and [12]]. Assessment of individual cardiomyocytes in H&E and reticulin-stained heart sections from 16-week-old SHP2^Y279C/+^ mice indicated that NSML hearts had enlarged individual cardiomyocytes, with increased overall cell surface area, that was normalized in response to the AKT inhibitor treatment of these mice (Fig 2A and 2C).

### ARQ 092 treatment improves cardiac function in NSML mice

To determine the effects of ARQ 092 on cardiac function, we conducted echocardiographic analysis of SHP2^+/+^ and SHP2^Y279C/+^ littermates, either in the presence of orally administered vehicle or ARQ 092 (100mg/kg/day), at 12, 14, and 16 weeks of age (Table 1, S2 Fig). By 12 weeks of age, as previously reported [11], SHP2^Y279C/+^ mice showed significant left ventricular hypertrophy, as indicated by decreased chamber dimension and increased posterior wall thickness compared with those of littermate controls; hypertrophy in these mice continued to progress over the 4 week time period (Table 1, S2 Fig). Treatment of the SHP2^Y279C/+^ mice with ARQ 092 normalized the HCM phenotype as early as 2 weeks following treatment, with levels comparable to those in SHP2^+/+^ at this time point (Table 1, S2 Fig). Interestingly, we also observed an increased fractional shortening, consistent with hypertrophy and increased contractility, in vehicle-treated NSML hearts; this was not apparent in ARQ 092-treated mice (Table 1), suggesting cardiac functional improvement. No functional changes or differences were observed in hearts of SHP2^+/+^ mice, whether treated with vehicle or ARQ 092 (Table 1, and S2 Fig).

**Table 1:** Anatomic and functional parameters, as assessed by echocardiography, in WT and NSML mice starting at 12 weeks of age and treated for the indicated length of time with vehicle or ARQ 092.

	Wildtype					SHP2 ^Y279C/+^				
	Baseline	Vehicle		ARQ092		Baseline	Vehicle		ARQ092	
**Age (weeks)**	12	14	16	14	16	12	14	16	14	16
**Tx (weeks)**	0	2	4	2	4	0	2	4	2	4
**IVSd (mm)**	1.09±0.04	1.01±0.04	1.10±0.06	1.18±0.06	1.10±0.06	1.19±0.05	1.20±0.06	1.38±0.06	1.03±0.03	1.19±0.10
**IVSs (mm)**	1.82±0.04	1.78±0.04	1.76±0.08	1.83±0.05	1.74±0.04	2.09±0.22	1.92±0.04	2.24±0.25	1.90±0.36	1.94±0.04
**LVIDd (mm)**	1.91±0.06	1.99±0.15	1.94±0.14	1.83±0.09	1.88±0.11	1.61±0.07*	1.75±0.06	1.69±0.05^	2.27±0.10	2.07±0.10#
**LVIDs (mm)**	0.41±0.03	0.42±0.04	0.43±0.08	0.41±0.03	0.43±0.05	0.27±0.02	0.27±0.04	0.33±0.01	0.37±0.07	0.36±0.06
**LVPWd (mm)**	1.07±0.03	1.11±0.08	1.30±0.12	1.04±0.07	1.24±0.14	1.32±0.04*	1.32±0.12	1.48±0.06^	1.01±0.09	1.23±0.22#
**LVPWs (mm)**	2.03±0.07	2.19±0.05	2.35±0.16	2.25±0.07	2.22±0.12	2.08±0.06	2.09±0.16	2.33±0.09	2.21±0.03	2.31±0.16
**EDV (ml)**	0.02±0.00	0.02±0.00	0.02±0.04	0.02±0.00	0.02±0.00	0.01±0.00	0.01±0.04	0.01±0.00^	0.03±0.00	0.02±0.00#
**EF (%)**	87.83±0.23	89.25±1.79	89.22±1.72	87.64±0.20	87.39±0.50	92.23±1.56	93.06±4.37	96.88±2.22	88.19±.29	88.21±0.35
**SV (ml)**	0.02±0.00	0.02±0.00	0.02±0.00	0.02±0.00	0.02±0.00	0.01±0.00	0.01±.00	0.01±0.00^	0.03±.00	0.02±.00#
**FS (%)**	70.47±1.22	70.95±2.32	71.11±2.56	68.79±0.84	68.14±2.53	77.76±1.96*	85.04±1.68	80.39±0.78^	74.88±2.25	73.31±2.36
**Heart rate**	670±10	673±30	680±13	672±15	673±31	668±10	682±28	714±18	673±13	697±25

Note: Baseline measurements are at 12 weeks of age. Mice were treated with either vehicle or ARQ 092 and then measured for cardiac function at 2 and 4 weeks of treatment. Mice were all sacrificed at 16 weeks of age. Data represent mean ± SEM. *, p<0.05, SHP2^+/+^ baseline vs. SHP2^Y279C/+^ baseline; ^, p<0.05, SHP2^+/+^ vehicle vs. SHP2^Y279C/+^ vehicle at 4 weeks; #, p<0.05, SHP2^Y279C/+^ vehicle at 4 weeks vs. SHP2^Y279C/+^ ARQ 092 at 4 weeks, where P values were derived from 2-way ANOVA on ranked data, with Bonferroni post hoc test when ANOVA was significant; n = 3–7 mice/group. IVSd, intraventricular septal diameter in diastole; IVSs, intraventricular septal diameter in systole; LVIDd, left ventricular chamber dimension in diastole; LVIDd, left ventricular chamber dimension in systole; LVPWd, left ventricular posterior wall thickness in diastole; LVPWs, left ventricular posterior wall thickness in systole; EDV, end diastolic volume; EF%, ejection fraction; SV, stroke volume; FS%, fractional shortening.

**Fig 3:**
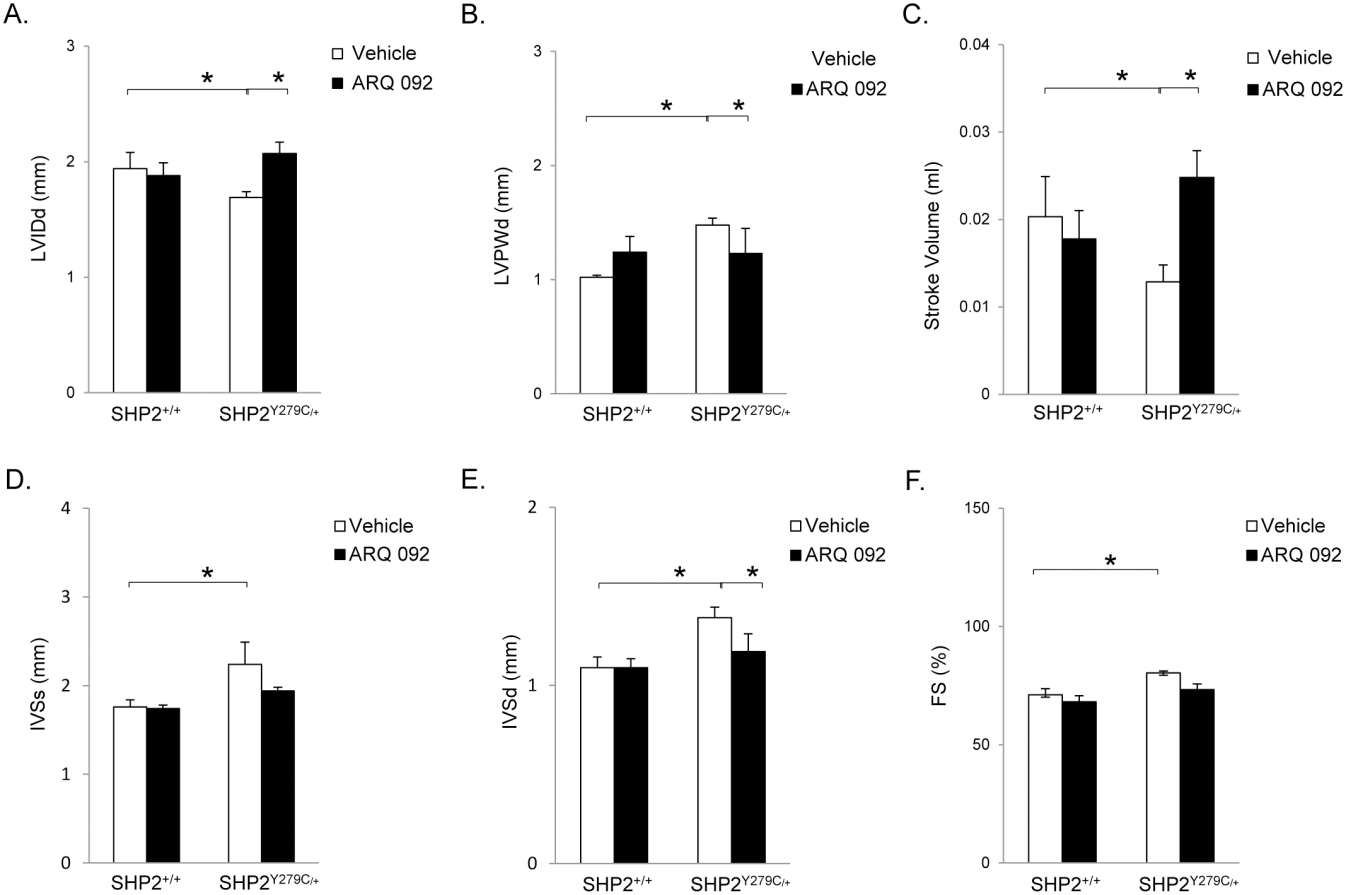
SHP2^Y279C/+^ hearts treated with ARQ 092 have improved cardiac function. Echocardiography was used to analyze the cardiac function in SHP2^+/+^ and SHP2^Y279C/+^ mice, either vehicle or ARQ 092 treated for 4 weeks, starting at 12 weeks of age. (A) LVIDd, (B) LVPWd, (C) SV (D) IVSs, (E) IVSd, and (F) FS% parameters were measured Data represent mean ± SEM. *P < 0.05, where P values were derived from from 2-way ANOVA on ranked data, with Bonferroni post hoc test when ANOVA was significant; n = 3–7 mice/group. LVIDd, left ventricular chamber dimension in diastole; LVPWd, left ventricular posterior wall thickness in diastole; SV, stroke volume; IVSs, intraventricular septal diameter in systole; IVSd, intraventricular septal diameter in diastole; FS%, fractional shortening.

Importantly, after 4 weeks of drug treatment, specific effects of the drug were observed in the cardiac functional parameters (Fig 3, S3 Fig). Chamber dimension (LVIDd) was increased (Fig 3A) and posterior wall thickness (LVPWd) was decreased (Fig 3B) in hearts from SHP2^Y279C/+^ mice treated with the drug, as compared to hearts from SHP2^Y279C/+^ vehicle-treated mice. Intraventricular septal dimension (IVSd), stroke volume (SV), and end-diastolic volume (EDV) from SHP2^Y279C/+^ drug-treated mice were also normalized in response to ARQ 092 treatment (Fig 3C, 3D and 3E), as compared to vehicle-treated controls. Interestingly, we observed an increase in fractional shortening (FS%) over time in the SHP2^Y279C/+^ mice, a compensatory effect that usually occurs in response to a pathological stimulus; this effect was not apparent in the SHP2^Y279C/+^ mice treated with the ARQ 092 drug (Fig 3F), suggesting functional improvement upon treatment with ARQ 092.

## Discussion

SHP2^Y279C/+^ mice have short stature, craniofacial dysmorphia, skeletal abnormalities, as well as pathological cardiac hypertrophy [11,12]. Biochemically, SHP2^Y279C/+^ mutant hearts have elevated AKT/mTOR activity, and we have previously shown that inhibiting mTOR with intraperitoneally-injected rapamycin could completely normalize and reverse the NSML cardiac defects, both *in vitro* and *in vivo* [11]. Here, we sought to determine whether inhibition upstream of mTOR, at the level of AKT, could similarly ameliorate the effects of hypertrophy in SHP2^Y279C/+^ mice. Moreover, as the ArQule allosteric AKT inhibitor, ARQ 092, is orally bioavailable, potent and selective, use of this inhibitor may be more readily tolerated and accepted by patients for treatment of NSML-associated cardiac hypertrophy.

Our results indicate that inhibition of AKT by ARQ 092 reduces hypertrophy in SHP2^Y279C/+^ mice, as early as 2 weeks following treatment, with normalized effects on the posterior wall thickness and chamber dimensions of treated SHP2^Y279C/+^ mouse hearts. Moreover, treatment with the ARQ 092 compound reduced the elevated fractional shortening (FS%) seen in the SHP2^Y279C/+^ vehicle-treated mice, an effect often associated with pathological compensatory hypertrophy, therefore suggesting that the inhibitor prevents the onset of this pathological event.

Importantly, we observed an overall decrease in overall heart weight in treated SHP2^Y279C/+^ mice; however, we did not see significant improvement in HW/BW ratios. In addition, we measured HW/TL ratios. We have previously found that bone structure/body length ratios are disproportionate in NSML mice, despite significant decreases in body weight [12]. Hereto, we found that tibia lengths in SHP2^Y279C/+^ mice are similar to SHP2^+/+^. As such, while we see a trending improvement in HW/TL ratios, we don’t reach significance. While we did not observe any other non-cardiac specific effects of the inhibitor in treated mice, we found that mice treated with ARQ 092 had reduced body weight, at least in the first two weeks of treatment, possibly due to administration by oral gavage, and/or to the direct use of the AKT inhibitor itself. Considering the significant involvement of AKT in several important biological processes, including adipogenesis and metabolic function, it is possible that AKT inhibition does indeed affect weight gain [17]. This effect may also contribute to the lack of significance in the physiological parameters for HW/BW ratios. Alternatively, it is possible that the 4 week time period in which this study was conducted is not sufficient to see overall improvement in HW/BW or HW/TL measures.

Additional evidence for positive effects of AKT/mTOR inhibition has been demonstrated. Previous pharmacological studies have shown that treatment of cardiomyocytes with an AKT inhibitor could prevent the hypertrophic response evoked by stimulation with various agonists [18–21]. Inhibition of AKT/mTOR signaling also attenuates or reverses pressure overload-associated cardiac hypertrophy [19,22,23], supporting our findings that there is a potential regulatory role for AKT/mTOR in pathological HCM in NSML [11,12].

In summary, our data support the conclusion that excessive AKT/mTOR leads to the development and maintenance of HCM in NSML, and that ARQ 092, a potent, selective, and orally bioavailable AKT inhibitor, can reverse hypertrophic cardiomyopathy in SHP2^Y279C/+^ mice. Although these findings hold promise for the treatment of hypertrophy in NSML, additional experiments, including use of an enlarged sample size, an optimization of dose and treatment duration, a measure of possible adverse effects, inhibitor effects on mortality or hemodynamics, and/or an assessment of the impact of inhibition of AKT itself, will be required to fully assess the clinical value of this inhibitor on this, and perhaps other, more common, congenital hypertrophy disorders that affect the AKT/mTOR signaling pathway.

## Materials and methods

### Histology

Hearts for morphometry and histochemistry were flushed with PBS, perfusion fixed in formalin or Bouin’s reagent, and paraffin embedded. Sections (5 μm) were stained with H&E, Masson-Trichrome, or reticulin at the Rodent Histopathology Core at Harvard Medical School. Crosssectional length, width, and area of cardiomyocytes with centrally located nuclei (to ensure the same plane of sectioning) were measured using ImageJ 1.41 software (developed by Wayne Rasband; http://rsbweb.nih.gov/ij/). Three individual samples were analyzed for each genotype, with 200–500 cells measured in each.

### Echocardiography

Transthoracic echocardiography was conducted on nonanesthetized animals as described previously [11,12], with a 13-MHz probe (Vivid 7, GE Medical Systems) or VisualSonics Vevo 770 high-frequency ultrasound rodent imaging system. GE Medical Systems or VisualSonics Vevo 770 software was used for data acquisition and subsequent analysis. Hearts were imaged in the 2-dimensional parasternal short-axis view, and an M-mode echocardiogram of the mid-ventricular region was recorded at the level of the papillary muscles. Calculations of cardiac anatomic and functional parameters were carried out as described previously [11,12].

### Biochemical analyses

Whole hearts from SHP2^+/+^ or SHP2^Y279C/+^ mice were dissected, perfused in PBS, and immediately frozen in liquid N2. Whole-cell lysates were prepared by homogenizing the tissue in radioimmunoprecipitation (RIPA) buffer (25 mmol/l Tris-HCl [pH 7.4], 150 mmol/l NaCl, 0.1% SDS, 1% NP-40, 0.5% sodium deoxycholate, 5 mmol/l EDTA, 1 mmol/l NaF, 1 mmol/l sodium orthovanadate, and a protease cocktail) at 4°C, followed by clarification at 14,000 g. Proteins were resolved by SDS-PAGE and transferred to PVDF membranes. Immunoblots were subsequently performed, following the manufacturer’s directions, with anti-AKT and anti-SHP2 antibodies (Santa Cruz Biotechnology Inc.); anti-phospho-S6RP, anti-phospho-PRAS40, anti-phospho-Akt473, anti-S6RP, and anti-PRAS40 antibodies (Cell Signaling Technology); and anti-GAPDH (Millipore). Bands were visualized with enhanced chemiluminescence and quantified by densitometry (developed by Wayne Rasband; ImageJ 1.41 software, http://rsbweb.nih.gov/ij/).

### Mice

SHP2^Y279C/+^ mice were described previously [11,12], and are now available from Jackson Laboratory as Stock No. 026759. Only male progeny were used for the experiments herein and all mice were maintained on outbred C57BL6/J backgrounds, backcrossed for more than 10 generations. All animal studies were approved by the Beth Israel Deaconess Medical Center IACUC Committee and performed in accordance with IACUC standards.

### *In vivo* AKT inhibitor studies

ARQ 092(ArQule, Inc) was dissolved in 0.01M phosphoric acid (vehicle) at a concentration of 20 mg/ml and filter sterilized. Either vehicle or ARQ 092 (100 mg/kg body weight) was then daily administered by oral gavage for 4 weeks. Administration began at 12 weeks of age (after established hypertrophy was indicated), and continued for 4 weeks, until the mice reached 16 weeks of age. As controls, SHP2^+/+^ and SHP2^Y279C/+^ mice were treated with vehicle alone.

### Statistics

All data are expressed as mean ± SEM. Statistical significance was determined using 2-tailed Student’s t test and 2-way ANOVA on ranked data, with Bonferroni post hoc test when ANOVA was significant; n = 3–7 mice/group. For all studies, values of P < 0.05 were considered statistically significant.

## Supporting information

S1 Fig Treatment with ARQ 092 reduces cardiac hypertrophy and normalizes heart size in NSML. (A). Heart weight to body weight ratio; (B) Heart weight to tibia length ratio; (C) Body weight; and (D) Tibia length of 16 week-old SHP2^+/+^ and SHP2^Y279/+^ littermates, either in the presence of vehicle or ARQ 092 AKT inhibitor for 4 weeks. Data represent mean ± SEM. *P < 0.05, where P values were derived from 2-way ANOVA on ranked data, with Bonferroni post hoc test when ANOVA was significant; n = 3–7 mice/group.
(TIF)

S2 Fig Representative echocardiography for ARQ 092 treated NSML mice. Representative echocardiographs of 16 week-old SHP2^+/+^ and SHP2^Y279C/+^ mice, at baseline, at 2 weeks, and at 4 weeks of treatment with either vehicle or ARQ 092. Baseline measurements began at 12 weeks of age and continued for 4 weeks, until 16 weeks of age.
(TIF)

S3 Fig SHP2^Y279C/+^ hearts treated with ARQ 092 have improved cardiac function. (dot plot analysis). Echocardiography from individual mice was used to analyze the cardiac function in SHP2^+/+^ and SHP2^Y279C/+^ mice, either vehicle or ARQ 092 treated for 4 weeks, starting at 12 weeks of age. (A) LVIDd, (B) LVPWd, (C) SV (D) IVSs, (E) IVSd, and (F) FS% parameters were measured Data represent mean ± SEM. *P < 0.05, where P values were derived from from 2-way ANOVA on ranked data, with Bonferroni post hoc test when ANOVA was significant; n = 3–7 mice/group. LVIDd, left ventricular chamber dimension in diastole; LVPWd, left ventricular posterior wall thickness in diastole; SV, stroke volume; IVSs, intraventricular septal diameter in systole; IVSd, intraventricular septal diameter in diastole; FS%, fractional shortening.
(TIF)
