# Supplementary figures and images for: In vivo efficacy of the AKT inhibitor ARQ 092 in Noonan Syndrome with multiple lentigines-associated hypertrophic cardiomyopathy
Source: PLoS One. 2017 Jun 5;12(6):e0178905. doi: 10.1371/journal.pone.0178905 (PMC5459472; doi:10.1371/journal.pone.0178905)

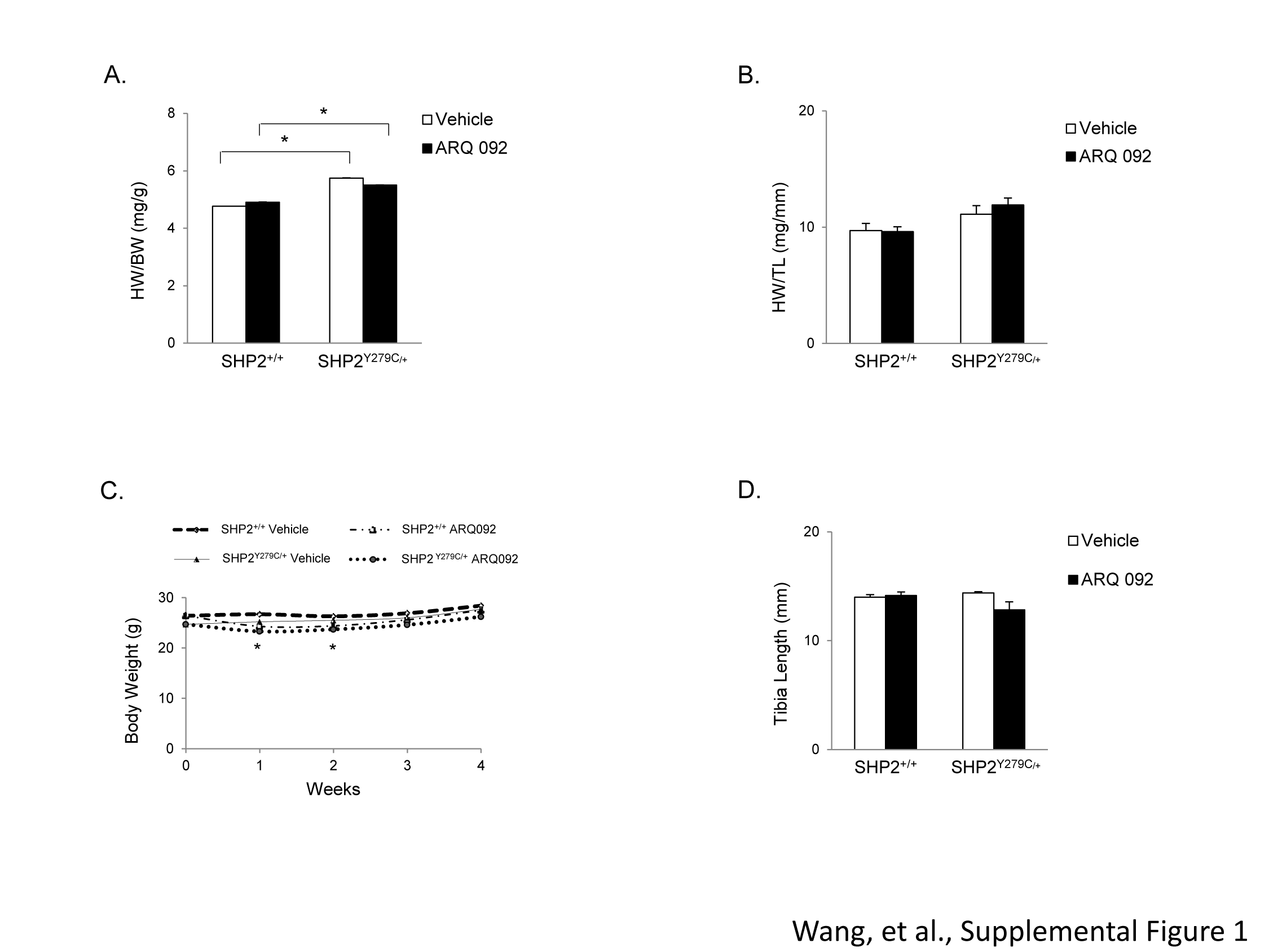

Supplement: S1 Fig — (A). Heart weight to body weight ratio; (B) Heart weight to tibia length ratio; (C) Body weight; and (D) Tibia length of 16 week-old SHP2+/+ and SHP2Y279/+ littermates, either in the presence of vehicle or ARQ 092 AKT inhibitor for 4 weeks. Data represent mean ± SEM. *P < 0.05, where P values were derived from 2-way ANOVA on ranked data, with Bonferroni post hoc test when ANOVA was significant; n = 3–7 mice/group. (TIF) [file pone.0178905.s001.TIF]

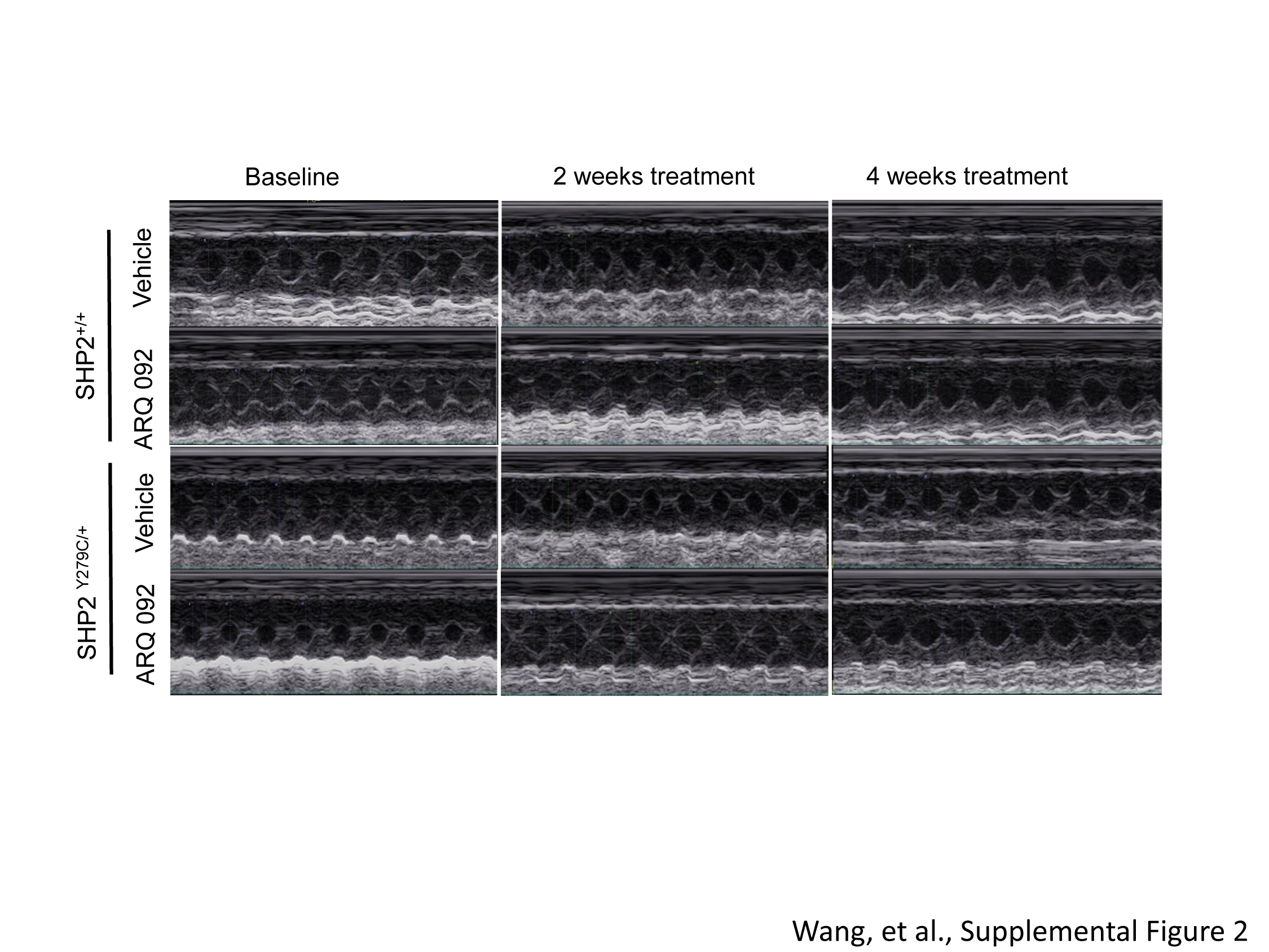

Supplement: S2 Fig — Representative echocardiographs of 16 week-old SHP2+/+ and SHP2Y279C/+ mice, at baseline, at 2 weeks, and at 4 weeks of treatment with either vehicle or ARQ 092. Baseline measurements began at 12 weeks of age and continued for 4 weeks, until 16 weeks of age. (TIF) [file pone.0178905.s002.TIF]

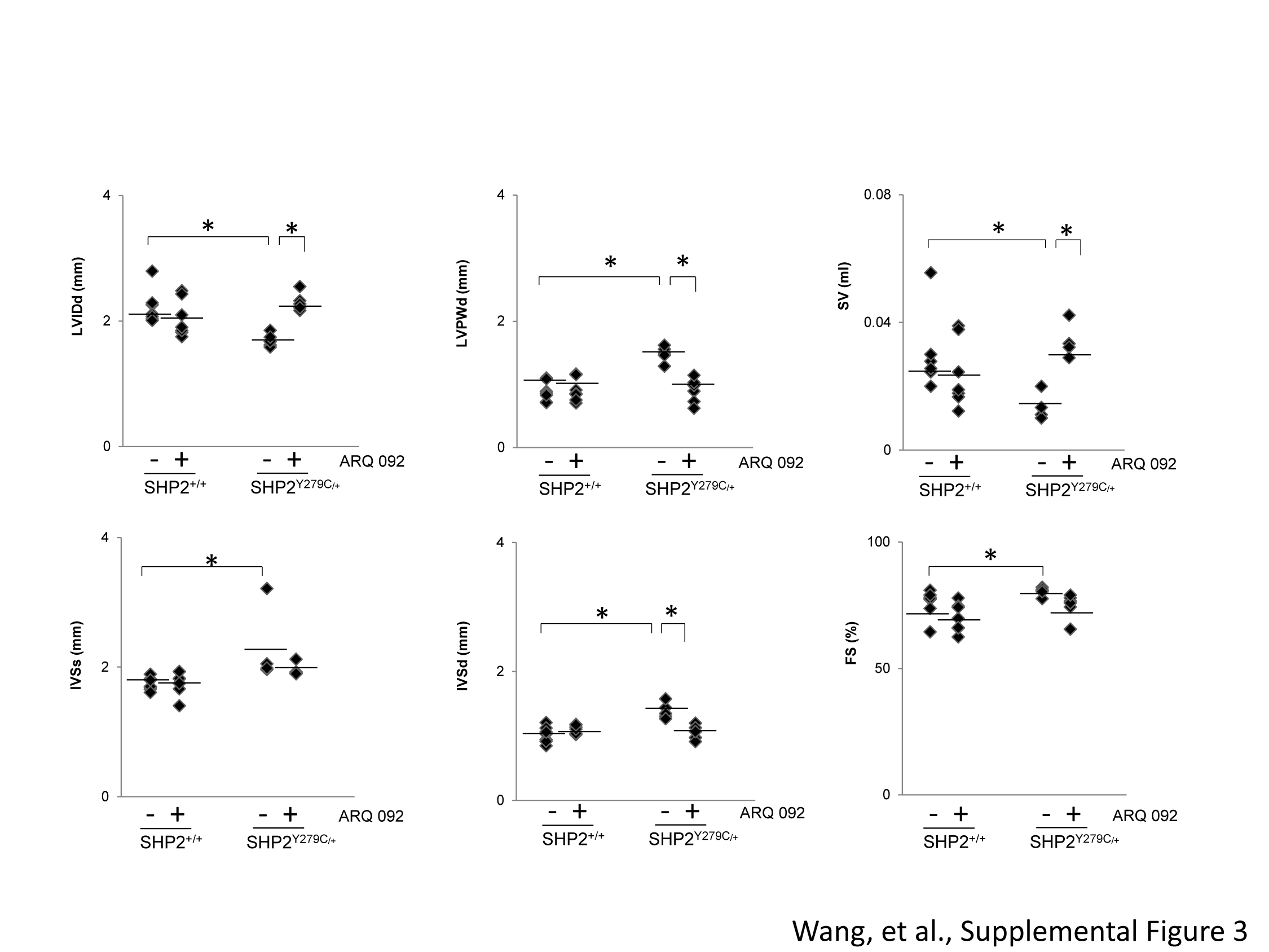

Supplement: S3 Fig — (dot plot analysis). Echocardiography from individual mice was used to analyze the cardiac function in SHP2+/+ and SHP2Y279C/+ mice, either vehicle or ARQ 092 treated for 4 weeks, starting at 12 weeks of age. (A) LVIDd, (B) LVPWd, (C) SV (D) IVSs, (E) IVSd, and (F) FS% parameters were measured Data represent mean ± SEM. *P < 0.05, where P values were derived from from 2-way ANOVA on ranked data, with Bonferroni post hoc test when ANOVA was significant; n = 3–7 mice/group. LVIDd, left ventricular chamber dimension in diastole; LVPWd, left ventricular posterior wall thickness in diastole; SV, stroke volume; IVSs, intraventricular septal diameter in systole; IVSd, intraventricular septal diameter in diastole; FS%, fractional shortening. (TIF) [file pone.0178905.s003.TIF]
